# Imaging of intracranial arterial disease: a comparison between MRI and unenhanced CT

**DOI:** 10.3389/fradi.2024.1338418

**Published:** 2024-02-15

**Authors:** Carlo Lucci, Ina Rissanen, Richard A. P. Takx, Anja G. van der Kolk, Anita A. Harteveld, Jan W. Dankbaar, Mirjam I. Geerlings, Pim A. de Jong, Jeroen Hendrikse

**Affiliations:** ^1^ Department of Radiology, University Medical Center Utrecht and Utrecht University, Utrecht, Netherlands; ^2^ Julius Center for Health Sciences and Primary Care, University Medical Center Utrecht and Utrecht University, Utrecht, Netherlands; ^3^ Department of General Practice, Amsterdam UMC, Location University of Amsterdam, Amsterdam, Netherlands; ^4^ Amsterdam Public Health, Aging & Later Life, and Personalized Medicine, Amsterdam, Netherlands; ^5^ Amsterdam Neuroscience, Neurodegeneration, and Mood, Anxiety, Psychosis, Stress, and Sleep, Amsterdam, Netherlands

**Keywords:** intracranial arterial calcification, stroke, vessel wall imaging MRI, CT scan (CT), magnetic resonance imaging (MRI)

## Abstract

**Background and purpose:**

Arterial calcifications on unenhanced CT scans and vessel wall lesions on MRI are often used interchangeably to portray intracranial arterial disease. However, the extent of pathology depicted with each technique is unclear. We investigated the presence and distribution of these two imaging findings in patients with a history of cerebrovascular disease.

**Materials and methods:**

We analyzed CT and MRI data from 78 patients admitted for stroke or TIA at our institution. Vessel wall lesions were assessed on 7 T MRI sequences, while arterial calcifications were assessed on CT scans. The number of vessel wall lesions, severity of intracranial internal carotid artery (iICA) calcifications, and overall presence and distribution of the two imaging findings were visually assessed in the intracranial arteries.

**Results:**

At least one vessel wall lesion or arterial calcification was assessed in 69 (88%) patients. Only the iICA and vertebral arteries (VA) showed a substantial number of both calcifications and vessel wall lesions. The other vessels showed almost exclusively vessel wall lesions. The number of vessel wall lesions was associated with the severity of iICA calcification (*p* = 0.013).

**Conclusions:**

The number of vessel wall lesions increases with the severity of iICA calcifications. Nonetheless, the distribution of vessel wall lesions on MRI and arterial calcifications on CT shows remarkable differences. These findings support the need for a combined approach to examine intracranial arterial disease.

## Introduction

Intracranial arterial disease is a major cause of ischemic cerebrovascular disease worldwide (1). While angiographic techniques are traditionally used to depict vessel lumen and identify stenosis, they are not suited to examine the arterial wall where the disease originates (2). Intracranial arterial disease can also manifest with non-stenosing lesions, and the composition of lesions can differ (3). Therefore, focusing exclusively on stenosis to define intracranial arterial disease can hamper progress in understanding this major disease (4).

Two imaging techniques are available that can provide information on the intracranial arterial wall. Unenhanced CT is the best method to visualize calcifications, while high-resolution intracranial vessel wall MR sequences are superior to CT angiography in visualizing vessel wall lesions, defined as focal or concentric thickenings of the arterial wall (5). These two imaging proxies of intracranial arterial disease are often used interchangeably to describe “intracranial atherosclerosis”. However, they depict various stages of arterial disease and sometimes different etiologies. Calcification occurs in atherosclerotic plaque formation, as well as in non-atherosclerotic diseases like Mönckeberg's calcification (6–8). In contrast, vessel wall lesions are due to infiltration of inflammatory mediators and lipid deposition in response to endothelial damage that marks the beginning of the atherosclerotic remodeling in most cases (9–12). Therefore, a clear understanding of intracranial arterial disease, as illustrated by these two imaging findings, is still required.

To shed light on the relation between intracranial calcifications and vessel wall lesions and their potential to depict intracranial arterial disease, the current study compared the presence and distribution of these two imaging findings in a cohort of patients with known cerebrovascular disease using unenhanced CT and 7 T MRI.

## Materials and methods

### Participants

For this study, we combined data from two clinical studies conducted at our institution: the Intracranial Vessel wall Imaging (IVI) study (62 patients) and the Posterior Intracranial Vessel wall Imaging (PIVI) study (16 patients). The IVI study included patients aged 18 years and older with a clinical history of acute ischemic stroke with a total or partial anterior circulation infarct or with a transient ischemic attack (TIA) which displayed symptoms related to the anterior circulation. The PIVI study included patients aged 18 years and older with ischemic stroke or TIA affecting the posterior cerebral circulation. Additional information on study inclusion and exclusion criteria, diagnostic criteria, and a flowchart of the study samples are provided in the Supplementary material. Both studies were approved by the review board of our institution, and written informed consent was obtained from all patients (10, 13).

As part of the protocol for both the IVI and PIVI study, all patients underwent 7 T MRI, including an intracranial vessel wall MRI sequence, within three months of onset of cerebrovascular symptoms. To undergo the 7 T MRI protocol only TIA patients or patients with minor to moderate stroke could be included as reflected by a NIH stroke scale at 1 week from the cerebrovascular event in the IVI study population of 5 points. Additionally, all patients admitted to our hospital with a possible cerebrovascular event underwent an unenhanced CT and a CT angiography as part of the clinical work-up for suspected cerebrovascular events. The CT examinations performed at our institution were retrieved and included in this study for analysis.

### MRI protocol

The MRI was conducted on a 7 T whole-body system (Philips Healthcare, Best, The Netherlands) with either a 16-channel (until May 2015) or 32-channel receive coil and volume transmit/receive coil for transmission (Nova Medical, Wilmington, MA, USA). Concerning the IVI study, for patients (*n* = 31) included until mid-July 2011 a reduced FOV 3D T1-weighted magnetization-prepared inversion recovery turbo spin echo (MPIR-TSE) sequence for visualization of the vessel wall was used, which excluded visualization of part of the basilar artery (BA) and vertebral arteries (VA). The scan parameters of this sequence have been described previously; in short, these were: FOV 220 × 180 × 13 mm^3^ in the transverse plane, acquired resolution 0.8 × 0.8 × 0.8 mm^3^, TSE factor+startup echoes 116, TR/TI/TE 6050/1770/80 ms, number of signal averages 2, and acquisition time ∼12 min (14). Due to technical developments, the remaining patients included in the IVI study (*n* = 31) and all patients in the PIVI study (*n* = 16) underwent a whole-brain 3D T1-weighted MPIR-TSE vessel wall sequence including the BA and VA. The scan parameters applied were: FOV 250 × 250 × 190 mm^3^ in sagittal plane, acquired resolution 0.8 × 0.8 × 0.8 mm^3^, TR/TI/TE 3952/1375/37 ms, TSE factor+startup echoes 168, number of signal averages 2, and acquisition time ∼11 min.

### CT protocol

Unenhanced CT and CTA images were obtained using either a Philips Brilliance 64-slice or iCT 256-slice CT scanner (Philips Healthcare, Best, The Netherlands) at 80–120 kVP and 250 mAs (adjusted based on patient body composition).

### MRI assessment

**Figure 1:**
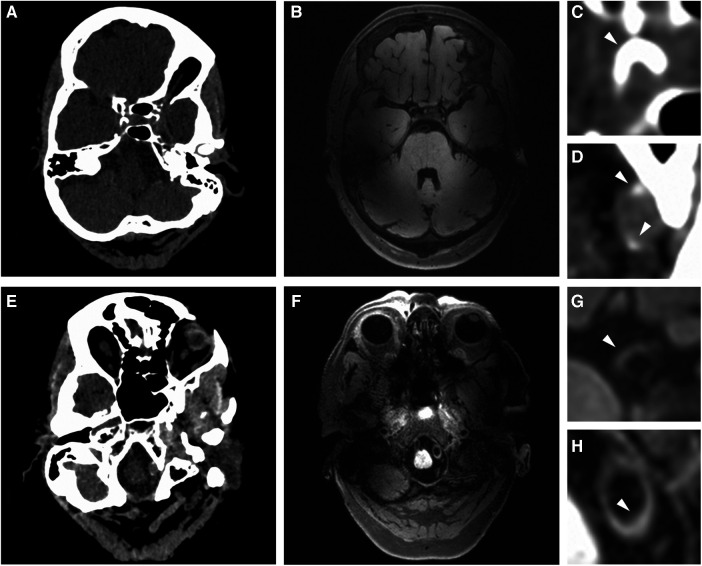
Imaging proxies of intracranial arterial disease. (**A**,**E**) Example of intracranial artery calcification on axial non-contrast head CT. (**B**,**F**) Example of vessel wall lesions on 7 T MRI. Zoomed images show: (**C**) a thick arterial calcification in the right ICA segment; (**D**) two calcified spots in the left VA segment; (**G**) a vessel wall lesion in the right ICA; (**H**) vessel wall lesion affecting a portion of the left VA.

The detailed scoring method of vessel wall lesions and measures of agreement between the raters have been previously described. Results of the interim analyses have been published (10, 13). The presence of one or more vessel wall lesions was assessed at 23 sites throughout the intracranial arterial circulation, which were subsequently grouped in 7 artery types (Supplementary material). According to previous literature, these segments can be divided according to their dimensions in large (MCA, ICA, VA and BA), medium (ACA and PCA) and small (PCOM) (15). The definition of a vessel wall lesion was either a focal or diffuse thickening of the vascular wall compared to the healthy contralateral or contiguous wall. The presence or absence of enhancement after administration of Gadolinium contrast agent was also assessed in the original studies. However, since vessel wall enhancement can be observed in atherosclerosis, as well as in other inflammatory and non-inflammatory diseases of the brain vasculature (i.e., vasculitis, amyloid deposition) (16, 17), we decided to remove this from our current analysis. Figure 1 shows an example of an intracranial vessel wall lesion detected on 7 T MRI.

### CT assessment

We analyzed unenhanced CT images of the cranium from the skull base to the vertex, reconstructed with a slice thickness ranging from 0.625 to 1 mm. The presence of calcification was evaluated as yes or no in the 23 sites of the intracranial arterial circulation by one neuroradiologist (JWD—more than 10 years of experience). The CTA images were used to aid in identifying the specific arteries. In addition, the severity of calcification in the intracranial internal carotid artery (iICA) was determined by one rater (CL—3 years of experience), according to the Woodcock score [15]. This scale distinguishes four levels of calcification severity according to thickness and continuity: absent, mild, moderate, and severe. The score is easy to learn and has been thoroughly validated, showing extremely high intra- and inter-rater reliability (18, 19). Therefore, we decided not to repeat the scoring between raters. Both raters were blinded to the clinical data and the vessel wall MRI assessment. The images were analyzed with free adjustment of the viewing plane via MPR and window level settings, using Sectra Workstation IDS7 19.3 (Sectra AB, Linköping, Sweden). Figure 1 displays an example of intracranial arterial calcifications detected on CT.

### Statistical analysis

First, we described the baseline patient characteristics of the study sample. Second, we assessed the overlap and differences in the distribution of vessel wall lesions and calcification for each arterial segment available, distinguishing absence of any imaging finding, exclusive vessel wall lesions, exclusive calcifications, or co-existence of the two. Third, to determine whether arterial disease as depicted by vessel wall lesions on MRI correlates with calcifications on CT, we used the Woodcock score in the ICA as a surrogate for the overall intracranial extent of calcifications. We then used the Jonckheere-Terpstra test (TJT) for ordered alternatives to test this relationship. Results were statistically significant at a *p*-value of ≤0.05. We used IBM SPSS Statistics (version 25 for Windows, IBM Corporation, Armonk, NY, USA) for the statistical analysis.

## Results

The mean age of the study sample (*n* = 78) was 60 ± 13 years, and 44% were women. Table 1 presents the baseline characteristics of the IVI and PIVI study patients. Of all patients, 58 (75%) had a stroke, 19 (24%) had a TIA, and 1 (1%) had another ischemic event. One-third of patients had received thrombolytic treatment. The median time between symptoms and the 7 T MRI examination was 6 days (10–90 percentile, 2–65), and 0 days for the CT examination (10–90 percentile, 0–5).

**Table 1:** Baseline characteristics of the study sample.

Baseline characteristics	All (*n* = 78)	IVI study (*n* = 62)	PIVI study (*n* = 16)
Age, years	59 (41–75)	60 (41–76)	57 (45–73)
Women, *n* (%)	34 (44)	31 (50)	3 (19)
Body mass index, kg/m^2^	25 (21–31)	26 (21–32)	25 (21–29)
Hyperlipidemia, *n* (%)	34 (44)	23 (37)	11 (67)
Diabetes mellitus, *n* (%)	10 (13)	8 (13)	2 (13)
Hypertension, *n* (%)	38 (49)	32 (52)	6 (38)
Smoking, *n* (%)	24 (31)	21 (34)	3 (19)
Stroke	58 (74)	44 (71)	14 (88)
TIA	19 (24)	17 (27)	2 (13)
Thrombolytic treatment	25 (33)	20 (33)	5 (33)
Time event to MRI, days	6 (2–65)	5 (1–43)	41 (18–95)
Time event to CT, days	0 (0–3)	0 (0–3)	0 (0–35)

Data are shown as number (percentage) or median (10–90 percentiles).

### Distribution of vessel wall lesions and arterial calcifications

The median number of vessel wall lesions per patient on MRI was 3 (range 0–23). Of patients, 89% (69/78) showed at least one arterial segment with a vessel wall lesion, and 89% (69/78) showed at least one calcified intracranial artery. ICA calcifications were mild in 22 patients, moderate in 38, and severe in 9 patients, while 9 patients presented with no calcifications at all. Supplementary material provides counts and percentages of vessel wall lesions on MRI and calcifications on CT for each artery type.

### Correlation between the imaging proxies

**Figure 2:**
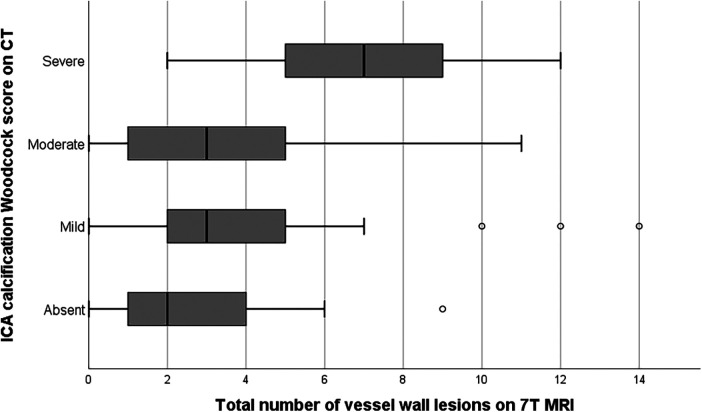
Bar graph showing the relation between vessel wall lesion burden and calcification severity in the internal carotid artery. Extreme values have been excluded from the image for better illustration of the trend of the association.

Sixty-two of the 78 patients presented with both vessel wall lesions and arterial calcifications: 14 patients showed calcification without any vessel wall lesion (*n* = 7) or vessel wall lesion(s) without calcification (*n* = 7); 2 patients did not show any presence of intracranial arterial disease. Figure 2 shows a significantly higher median burden of vessel wall lesions in patients with severe calcification of the iICA on the Woodcock score (TJT-statistic = 1.26, *z* = 2.48, *p* = 0.013). No statistical difference was noted between the burden and vessel wall lesions and absent, mild, and moderate levels of iICA calcification as assessed by the Woodcock score.

### Overlap and differences between the imaging proxies

**Figure 3:**
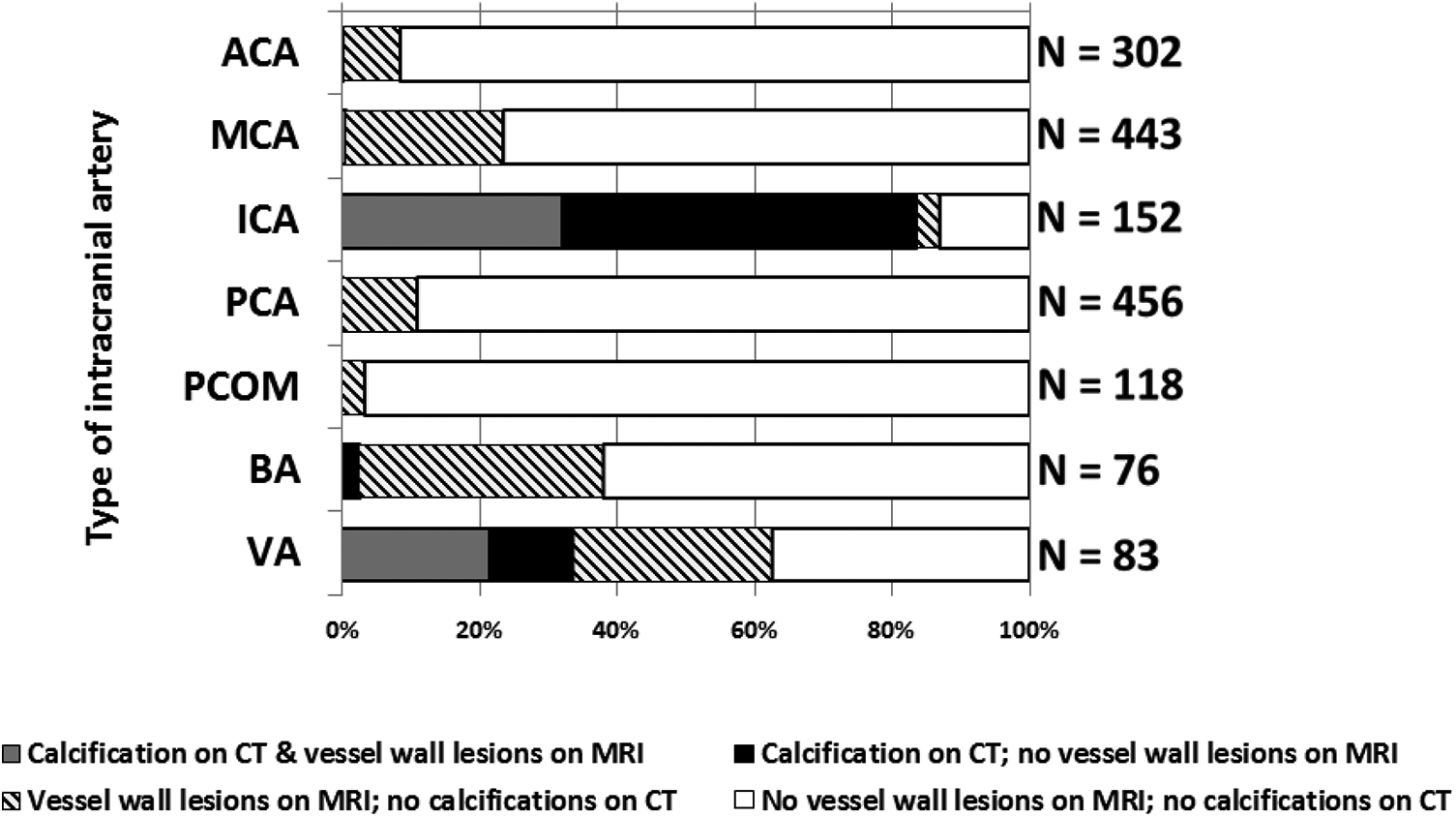
Graph showing the distribution of intracranial vessel wall lesions and arterial calcifications in 7 different arterial segments. Data from left and right segments are showed together. ACA, anterior cerebral artery; MCA, middle cerebral artery; PCA, posterior cerebral artery; PCOM, posterior communicating artery; BA, basilar artery; VA, vertebral artery. Left and right segments taken together (if applicable).

Figure 3 shows the minor overlap and major differences between the two imaging proxies of intracranial arterial disease. Only the iICA and the VA showed a substantial number of calcifications as well as vessel wall lesions while the other vessels (ACA, MCA, PCA, and PCOM) showed almost exclusively vessel wall lesions. 32% of the iICA arterial segments showed both calcifications and vessel wall lesions, 51% showed only calcification, and few segments (3%) showed exclusive vessel wall lesions. In 22% of the VA, both imaging proxies occurred together, 29% showed exclusively vessel wall lesions, and 12% manifested exclusive calcifications. 3% of the BA segments presented exclusive calcifications, while 57% showed only vessel wall lesions.

## Discussion

This study assessed the intracranial distribution of arterial calcifications on unenhanced CT and vessel wall lesions on 7 T MRI in patients with known cerebrovascular disease. We found major differences between the distribution of these imaging findings that are frequently used as intracranial atherosclerotic disease indicators and only a significant association of severe iICA calcification as defined by the Woodcock score and the overall burden of MRI vessel wall lesions.

### Calcifications vs. vessel wall lesions

The overall burden of vessel wall lesions in the intracranial arterial circulation is associated with the severity of iICA calcifications. Severe carotid artery calcification in stroke patients could help identify patients eligible for an MRI vessel wall examination in the clinical setting given the higher burden of non-calcified intracranial arterial disease these patients might carry. However, vessel wall pathology assessed with MRI showed a profoundly different distribution pattern compared to arterial calcification on unenhanced CT. Advanced atherosclerotic plaques (fibrous and calcified) affect mainly the large arteries of the brain (namely iICA, VA, BA, and MCA) while medium and small arteries (ACA, PCA, and PCOM) are more affected by early-stage atherosclerotic lesions (manifesting only as intimal thickening and fatty streaks).

The iICA showed calcification in 84% of cases vs. approximately 35% that showed vessel wall lesions, while in the VA approximately 30% of segments presented with calcifications vs. ca. 50% that had vessel wall lesions. The discrepancy between anterior and posterior circulation could be due to the reliability of MR vessel wall lesion assessment in the internal carotid artery, which has the lowest reliability due to its tortuous course and location at the skull base. Severe calcifications could also hamper evaluation of vessel wall thickness. Arterial wall lesions in the anterior brain vasculature could be left unidentified and cause underestimation of total disease burden.

### Distribution of imaging proxies of intracranial arterial disease

From previous literature, overall prevalence of arterial calcifications is highest in the iICA (∼60%) followed by the VA (∼20%), MCA (∼5%), and BA (∼5%), while the PCA and ACA are rarely affected (18). The distribution of arterial calcification in our study sample follows the same pattern, albeit with an overall higher frequency of calcifications due to the selection of patients with known cerebrovascular disease. Intracranial artery calcifications are more prevalent in stroke patients and are recognized as an independent risk factor for stroke (20–22). A study of patients with internal carotid artery stenosis ≥60% found that calcified atherosclerotic plaques in the carotid artery were 21 times less likely to be symptomatic compared to non-calcified plaques, even after adjusting for traditional cardiovascular risk factors (5, 23). This evidence suggests that the presence of calcification alone is not sufficient for accurately predicting cerebrovascular disease.

Intracranial vessel wall imaging has seen a recent growth in popularity for the characterization of vessel wall pathology (24). The prevalence of at least one intracranial vessel wall lesion was estimated to be around 40% in a recent population-based study focusing on vessel wall MRI (25). In the current study, performed in patients with known history of ischemic stroke or TIA, we found an overall prevalence of 89%. This may be due to the use of 7 T MRI data, which has shown to be more sensitive to subtle thickening of the intracranial vessel wall (26). Additionally, patients with known cerebrovascular disease were selected, which increases the likelihood of a higher burden of intracranial arterial disease (27). Therefore the use of vessel wall MRI is certainly valuable for secondary prevention of ischemic events. However, the prognostic value of vessel wall lesions assessment *in vivo* in still asymptomatic patients has not yet been established.

### Strengths and limitations

This study is among the first to investigate two imaging proxies of intracranial arterial disease thanks to the availability of both clinically acquired unenhanced CT and vessel wall MRI data in patients with known cerebrovascular disease. While recent 3 T studies also showed the ability to depict vessel wall lesions with high accuracy, 7 T MRI proved to be more sensitive to small vessel wall lesions due to its higher spatial resolution and contrast-to-noise ratio (27, 28).

There are several limitations to the current investigation that deserve mention. Our analyses were performed on a relatively small group of stroke/TIA patients with good physical status, which may not reflect the severe stroke population. Nonetheless, including healthier patients would only dilute the correlation we found, not invalidate our results. Additionally, obtaining histological data in patients with intracranial atherosclerosis is virtually impossible, which limits the possibility of confirming the nature of our imaging findings. We focus only on intracranial arteries without providing information about extracranial arterial disease which is known to be a significant risk factor for stroke (29). However, previous research shows that the risk factors for intracranial and extracranial arterial disease are different (30) and therefore might depict different causes of arterial disease. Future studies are needed to assess the distribution and the relation between these imaging findings in intracranial and extracranial arteries.

## Conclusion

We discovered a significant relationship between the severity of arterial calcifications and vessel wall lesions in intracranial arteries. That being said, we also noticed significant differences in the distribution of intracranial arterial calcification on CT scans and arterial lesion assessments on vessel wall MRI. The differences suggest that these two imaging findings might depict different types of intracranial diseases, in light of the recent findings of intracranial non-atherosclerotic medial calcification, or various stages of atherosclerotic disease. Therefore, a combination of both imaging modalities could provide complementary information to non-invasively depict the real burden of intracranial arterial disease considering its relation with an increasing risk of stroke.

## Data Availability

The data analyzed in this study is subject to the following licenses/restrictions: Patient data. Requests to access these datasets should be directed to Jeroen Hendrikse, j.hendrikse@umcutrecht.nl.
