# Supplementary material for: Imaging of intracranial arterial disease: a comparison between MRI and unenhanced CT
Source: Front Radiol. 2024 Feb 15;4:1338418. doi: 10.3389/fradi.2024.1338418 (PMC10902099; doi:10.3389/fradi.2024.1338418)
Supplement: Supplementary file 1 [file Datasheet1.pdf]

SUPPLEMENTARY MATERIAL

This data was provided by the authors to give readers additional information about the work described in: **“Imaging of Intracranial Arterial Disease: A Comparison Between MRI and Unenhanced CT”**

Table of contents

1. **Supplementary methods**
2. **Supplementary tables**

1. Supplementary methods

Participants

The Intracranial Vessel wall Imaging (IVI) study (NTR2119, www.trialregister.nl), started in December 2009, and the Posterior Intracranial Vessel wall Imaging (PIVI) study (NTR5688) started in November 2013.

The IVI study included 96 patients with a clinical history of acute ischemic stroke with a total/partial anterior circulation infarct or with a transient ischemic attack (TIA) which displayed symptoms related to the anterior circulation. Exclusion criteria was hemorrhage detected on the initial CT scan of the brain. The final diagnosis of the underlying cause of the stroke was determined using the Stop Stroke Study Trial of Org 10172 in Acute Stroke Treatment (SSS-TOAST) classification criteria and a comprehensive clinical examination. For further information see the article: ′′**Van der Kolk AG, Zwanenburg JJM, Brundel M, et al. Distribution and natural course of intracranial vessel wall lesions in patients with ischemic stroke or TIA at 7.0 tesla MRI. Eur Radiol. 2015;25(6):1692-1700. doi:10.1007/s00330-014-3564-4′′.**

The PIVI study included 25 patients with ischemic stroke or TIA affecting the posterior cerebral circulation. Exclusion criteria were contraindications for undergoing MR imaging such as claustrophobia, the presence of specific metal objects in or on the body, pregnancy, known allergy to gadolinium-containing contrast agent, or severe renal impairment. Additionally, patients who had a transient ischemic attack (TIA) or ischemic stroke caused by a surgical or interventional procedure, previous surgery or endovascular therapy related to the vertebrobasilar region were also excluded from the study. For further details see the article: **′′ Harteveld AA, Van Der Kolk AG, Van Der Worp HB, et al. Detecting Intracranial**

Vessel Wall Lesions with 7T-Magnetic Resonance Imaging: Patients with Posterior Circulation Ischemia Versus Healthy Controls. Stroke. 2017;48(9):2601-2604. doi:10.1161/STROKEAHA.117.017868''.

Flowchart of the study sample

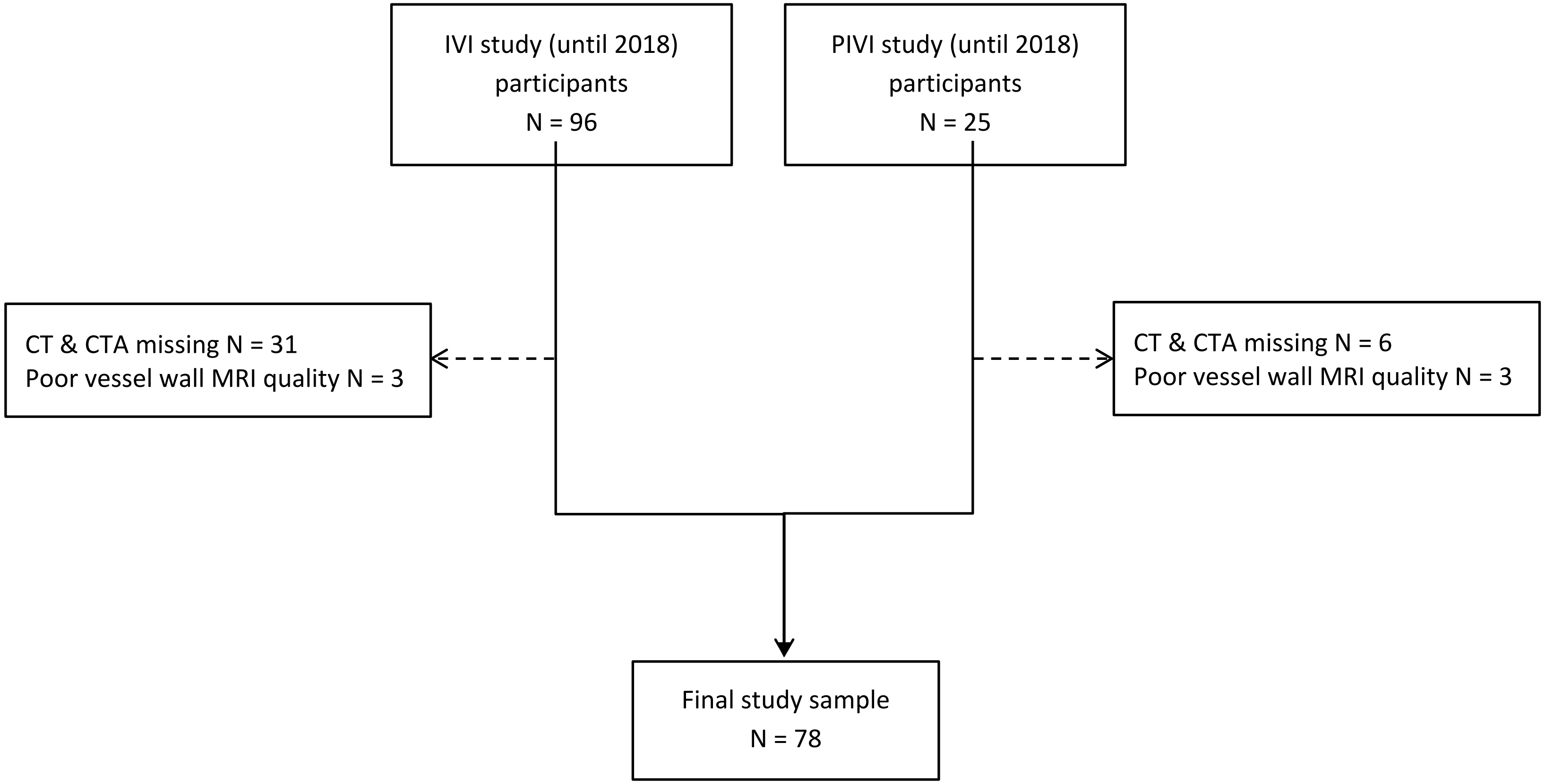

3. **Supplementary tables**

Artery	Segment*	Number and % of arteries showing vessel wall lesions	Number and % of arteries showing arterial calcification
**ACA**	*A1*	14/151 (9.3)	1/156 (0.6)
	A2	11/151 (7.3)	0/156 (0.0)
**MCA**	*M1*	40/152 (26.3)	1/156 (0.6)
	M2	26/140 (18.6)	0/156 (0.0)
	Bifurcation ICA-M1	38/151 (25.2)	2/156 (1.3)
**ICA**	*ICA*	54/152 (35.5)	131/156 (84.0)
**PCA**	*P1*	18/153 (11.8)	1/155 (0.6)
	P2	24/152 (15.8)	0/156 (0.0)

	Bifurcation P1-P2	7/152 (4.6)	0/156 (0.0)
**PCOM**	-	4/120 (3.3)	0/154 (0.0)
**BA**	-	27/76 (35.5)	3/156 (1.9)
**VA**	-	42/83 (50.6)	44/156 (28.2)
	Total	305/1633 (18.7)	183/1869 (9.8)

Artery types and counts and percentages per segment of vessel wall lesions on MRI and arterial calcifications: denominators reflect number of segments suitable for assessment. **ACA** = anterior cerebral artery; **MCA** = middle cerebral artery; **PCA** = posterior cerebral artery; **PCOM** = posterior communicating artery; **BA** = basilar artery; **VA** = vertebral artery. * Left and right taken together (when applicable)
